# Potential protein post-translational modification in ERp57: A phenotype marker for male fertility

**DOI:** 10.4103/0974-1208.74158

**Published:** 2010

**Authors:** Viroj Wiwanitkit

**Affiliations:** Wiwanitkit House, Bangkhae, Bangkok, Thailand

**Keywords:** Post-translational modifications, ERp57, male, infertility

## Abstract

**BACKGROUND:**

In protein expression, post-translational modification is an important process. It is also an important process in human reproductive science. ERp57 is a molecule that is mentioned for post-translational modification. ERp57 is a component of human sperm acrosome proteins. However, the data on post-translational modifications of ERp57 is limited.

**AIM:**

The aim of this work is to assess potential protein post-translational modifications in ERp57 protein.

**SETTINGS AND DESIGN:**

A descriptive computational bioinformatics study.

**MATERIALS AND METHODS:**

In this work, potential protein post-translational modifications in ERp57 protein were assessed via a standard bioinformatics technique.

**STATISTICAL ANALYSIS USED:**

Bioinformatics analysis.

**RESULTS:**

There are three post-translational modifications within ERp57 from bioinformatics analysis.

**CONCLUSION:**

This new knowledge can be useful for better realization on molecular process of male infertility.

## INTRODUCTION

In protein expression, post-translational modification is an important process.[1] This process plays important roles in further mechanisms of cell regulation.[1] It is also an important process in human reproductive science. ERp57 is a molecule that is mentioned for post-translational modification.[2] ERp57 is a component of human sperm acrosome proteins.[2] It plays an important role in gamete fusion and can be used as a novel phenotype marker for male infertility.[2]

Post-translational modification of ERp57 can be seen during sperm capacitation.[2] However, the data on post-translational modifications of ERp57 is limited. In this work, potential post-translational modifications in ERp57 protein were assessed via a standard bioinformatics technique.

## MATERIALS AND METHODS

The author uses standard reference, PubMed (www.pubmed.com), searching to directly obtain the sequence for ERp57. Then the derived sequence was used for further finding for potential protein post-translational modifications via a standard bioinformatics tool namely FindMod.[3] The operative parameters in this study for 1) tolerance; it was set to ±0.5 Dalton and 2) enzymes; they were as 2.1) trypsin, allowing for up to three missed cleavages, 2.2) methionine and tryptophan in oxidized form, and 2.3) cysteine in reduced form, with acrylamide adducts. The allowable resulted peptide mass in this work is bigger than 500 Dalton. The protocol for this work is the same as previously published protocol in referencing previous publications.[4,5]

## RESULTS

According to the searching, ERp57 could be derived. There are three post-translational modifications within Erp57 from bioinformatics analysis [Table 1].

**Table 1:** Potential post-translational modifications

Potential modification	Peptide	Position
METH-TRYP	K	62
METH-TRYP	K	79
METH-TRYP	K	356

## DISCUSSION

The underlying molecular pathogenesis of male infertility is of interest. The present research in human reproductive science focuses on the molecular biological process for male infertility. ERp57 is accepted as a possible biomarker for male infertility.[2] Accompanied with calnexin and calreticulin, ERp57 helps in regulating glycoprotein folding that is an important process in sperm functioning.[6] Basically, ERp57 acts as a chaperone for Ca2+ regulation within endoplasmic reticulum.[7–8] Hence, ERp57 is very important for any motile cells including spermatozoa. In conclusion, ERp57 plays an important role in sperm function and its help in regulating sperm motility is an important factor in male fertility.

Post-translational modification of ERp57 is mentioned in the literature. Focusing on mechanism involved in post-translational protein modification *in vivo*, the oxidation process is observed.[9] Molinari *et al*., reported that the endoplasmic-reticulum-resident oxidoreductases PDI and ERp57 were directly involved in disulfide oxidation and isomerization, and, together with the lectins calnexin and calreticulin, were central in glycoprotein folding in the endoplasmic reticulum of cells.[9] Of interest, the described process of oxidation is strongly relating to nitric oxide production that is important for sperm motility.[10]

In this article, the author successfully identified and reported three sites within the ERp57 molecule that is highly prone for phosphorylation post-translational modification. This is the basic research that can be the basic information for further studies. This new knowledge can be useful for better realization on molecular process of male infertility. It might open up new avenues for male infertility assessment. Nevertheless, it should also be noted that the work focuses mainly on prediction of post-translational modification, not the derivation of ERp57 or its function. Hence, further studies to assess those aspects to fulfill the information area are suggested.

However, some limitations of this work should be discussed. This work is a bioinformatics approach that still needs further verifications. Although the bioinformatics tool used in the manuscript is a standard one but there are also other available refined bioinformatics tools that can help in determining different post-translational protein modifications. Nevertheless, the reliability of the used technique in this work is already proved.[4–5] In addition, the results in this work can at least confirm the previous publication by Zhang *et al*.,[11] which clearly indicated that a protein phosphorylation modification is involved. Zhang *et al*., reported that “When the ERp57 expression was compared in capacitated and uncapacitated sperm, it was observed that all spots had the same molecular weight but differing pIs.”[11] This kind of shift is possible if there is a modulation in phosphorylation level but not glycosylation level. Glycosylation of any protein will change the molecular weight of the protein, which might not the same in case of ERp57. If one carries out bioinformatics analysis of ERp57 protein, several potential phopshorylation sites in the protein might be identified and the result in this work, several possible potential phopshorylation sites, is totally concordant. In additional, change of molecular weights and basic quantum energies due to observed amino acid changes might be the possible explanations for impact on the sperm function.

## References

[1] Witze ES, Old WM, Resing KA, Ahn NG (2007). Mapping protein post-translational modifications with mass spectrometry. Nat Methods.

[2] Zhang J, Wu J, Huo R, Mao Y, Lu Y, Guo X (2007). ERp57 is a potential biomarker for human fertilization capability. Mol Hum Reprod.

[3] Wilkins MR, Gasteiger E, Gooley AA, Herbert BR, Molloy MP, Binz PA (1999). High-throughput mass spectrometric discovery of protein post-translational modifications. J Mol Biol.

[4] Wiwanitkit V (2008). Potential protein post-translational modifications and find potential single amino acid substitutions in hepatitis B large envelope protein. Rev Esp Enferm Dig.

[5] Wiwanitkit V (2009). Prediction of potential protein post-translational modifications of the thioredoxin-1 molecule. Cardiovasc J Afr.

[6] Ellgaard L, Frickel EM (2003). Calnexin, calreticulin, and ERp57: teammates in glycoprotein folding. Cell Biochem Biophys.

[7] Corbett EF, Oikawa K, Francois P, Tessier DC, Kay C, Bergeron JJ (1999). Ca2+ regulation of interactions between endoplasmic reticulum chaperones. J Biol Chem.

[8] Vangheluwe P, Raeymaekers L, Dode L, Wuytack F (2005). Modulating sarco (endo) plasmic reticulum Ca2+ ATPase 2 (SERCA2) activity: cell biological implications. Cell Calcium.

[9] Molinari M, Helenius A (1999). Glycoproteins form mixed disulphides with oxidoreductases during folding in living cells. Nature.

[10] Lewis SE, Donnelly ET, Sterling ES, Kennedy MS, Thompson W, Chakravarthy U (1996). Nitric oxide synthase and nitrite production in human spermatozoa: evidence that endogenous nitric oxide is beneficial to sperm motility. Mol Hum Reprod.

[11] Zhang J, Wu J, Huo R, Mao Y, Lu Y, Guo X (2007). ERp57 is a potential biomarker for human fertilization capability. Mol Hum Reprod.

